# Early prediction of acute kidney injury following ICU admission using a multivariate panel of physiological measurements

**DOI:** 10.1186/s12911-019-0733-z

**Published:** 2019-01-31

**Authors:** Lindsay P. Zimmerman, Paul A. Reyfman, Angela D. R. Smith, Zexian Zeng, Abel Kho, L. Nelson Sanchez-Pinto, Yuan Luo

**Affiliations:** Northwestern University, Evanston, IL 60208 USA

**Keywords:** Physiological measurements, Acute kidney injury, Intensive care unit, Predictive modeling, Multivariate logistic regression, Random forest, Artificial neural networks

## Abstract

**Background:**

The development of acute kidney injury (AKI) during an intensive care unit (ICU) admission is associated with increased morbidity and mortality.

**Methods:**

Our objective was to develop and validate a data driven multivariable clinical predictive model for early detection of AKI among a large cohort of adult critical care patients. We utilized data form the Medical Information Mart for Intensive Care III (MIMIC-III) for all patients who had a creatinine measured for 3 days following ICU admission and excluded patients with pre-existing condition of Chronic Kidney Disease and Acute Kidney Injury on admission. Data extracted included patient age, gender, ethnicity, creatinine, other vital signs and lab values during the first day of ICU admission, whether the patient was mechanically ventilated during the first day of ICU admission, and the hourly rate of urine output during the first day of ICU admission.

**Results:**

Utilizing the demographics, the clinical data and the laboratory test measurements from Day 1 of ICU admission, we accurately predicted max serum creatinine level during Day 2 and Day 3 with a root mean square error of 0.224 mg/dL. We demonstrated that using machine learning models (multivariate logistic regression, random forest and artificial neural networks) with demographics and physiologic features can predict AKI onset as defined by the current clinical guideline with a competitive AUC (mean AUC 0.783 by our all-feature, logistic-regression model), while previous models aimed at more specific patient cohorts.

**Conclusions:**

Experimental results suggest that our model has the potential to assist clinicians in identifying patients at greater risk of new onset of AKI in critical care setting. Prospective trials with independent model training and external validation cohorts are needed to further evaluate the clinical utility of this approach and potentially instituting interventions to decrease the likelihood of developing AKI.

## Background

Acute kidney injury (AKI) is commonly seen in adults in the intensive care unit (ICU). AKI is one of the major diagnoses among ICU patients and a leading factor associated with a prolonged hospital stay and with subsequent morbidity or early mortality post discharge [1–4]. Acute renal failure is a complex disorder that presents itself in a variety of settings with clinical manifestations ranging from a minimal elevation in serum creatinine to anuric renal failure [5]. Unfortunately, the main biomarker of AKI, serum creatinine (SCr), is a late marker of injury, which delays diagnosis and treatment [6].

Previous studies highlight the importance of early recognition of AKI, as well as the association of AKI with increased mortality in hospitalized patients. As electronic health records (EHRs) become more prevalent, the increasing availability of comprehensive clinical databases provides the possibility of developing predictive models using data collected from thousands of patient encounters [5]. Efficacy of interventions often depends on the interplay between early identification of deterioration, the timing of interventions, and choice of interventions (e.g. selection of appropriate fluid resuscitation strategy, use of vasopressors, and administration of renoprotective agents). Multiple patient and healthcare delivery related risk factors have been shown as predictors of AKI in specific patient cohorts [7, 8]. However, correlations between these diverse set of risk factors across heterogeneous patient cohorts are much less understood, but critical for producing effective diagnostic and treatment guidelines of AKI [2, 3], Such guidelines often need a panel of demographic, clinical physiologic, and radiologic features in order to stratify patient cohorts for targeted treatment.

Currently, there is no mutually accepted definition of acute kidney dysfunction. Varying terms, such as acute renal failure, renal insufficiency, kidney injury, and renal impairment, and multiple definitions (e.g. percent or absolute increments of creatinine, or decrements of urine output) have been used previously [9]. The term acute kidney injury has gained momentum as the best nomenclature to replace acute renal failure because the spectrum of AKI is broad and includes varying degrees of severity.

We used the definition of AKI as described in the Kidney Disease Improving Global Outcomes (KDIGO) [10], in order to standardize the published diagnostic criteria. The diagnostic criteria are defined as an acute increase in the absolute level of serum creatinine of more than 0.3 mg/dl or 50% higher change in serum creatinine (SCr) from baseline within a 48-h period or decreased glomerular filtration rate (GFR) to less than 0.5 ml/kg/hour for more than six hours [9, 10]. These criteria were based on accumulating evidence that even small alterations in SCr are associated with dire consequences.

Many factors including nephrotoxic medications, insufficient effective circulating fluid volume, and intrinsic renal disease can cause or contribute to AKI [11, 12]. AKI can be diagnosed using any of several sets of diagnostic and classification criteria [9, 11, 13]. These diagnostic criteria are all based, in large part, on the commonly measured serum/plasma biomarker creatinine, whereby defined increases in creatinine occurring within a specific period of time (e.g. 24, 48 or 168 h) will be diagnostic of AKI. Thus, an accurate creatinine forecast, as we plan to develop in this study, should in turn enable prediction of AKI risk. In this study, we focus on predicting AKI using first-day measurements of a multivariate panel of physiologic variables, in order to elucidate early, subclinical deterioration of patient’s physiologic baselines that are predictive of AKI.

### Related work

Numerous previously published studies describe AKI prediction models using EHR data [14–24]. Most models had modest performance with area under the receiver operating curves (AUC) approximating 0.75. However, many studies focus on specific patient groups such as cardiac surgery patients, septic shock patients, and elderly patients, or focus on the validation of novel biomarkers. Less work has been performed for general intensive care populations despite the fact that ICU patients also have high risk of AKI. Many previous studies also have small patient population due to specific focus. In addition, there is still a gap between existing studies and the need to identify high-risk AKI patients as early as possible. The approaches and goals of this study differ from previously published reports in that it aims to address these questions by utilizing a large clinical database and building a predictive model that enables early AKI detection. Many prior AKI prediction models, while nonetheless clinically useful in many settings, i) rely on various static scoring algorithms, often including a limited set of features in part to facilitate human (offline) computation; ii) incorporate non-routine biomarkers (e.g. NGAL) in predictions; and/or iii) do not model temporal progression of clinical, laboratory and other predictive information, which has been shown to be effective for clinical predictive modeling [25]. As a result, many previously developed models are not optimally suited for clinical decision making that forecasts AKI in a general patient population. For example, a predictive model that incorporates a limited set of predictors and, in particular, a limited array of clinical interventions as predictors, could not identify the impact that changes in clinical care might have on AKI risk. Likewise, models that rely heavily on biomarkers that are not routinely tested would be unable to accurately screen for AKI risk in a general patient population. Our approach, in contrast, involves the careful modeling of a wide array of predictor data including clinical treatments and the temporal aggregation of predictor data. Including a wide array of predictors may permit the models to provide predictions that are more patient-specific and suitable for clinical scenario testing. In addition, our approach focuses on the early prediction of AKI on patients who do not meet AKI criteria on admission to the ICU, thus targeting a population that could benefit from early preventive strategies that can prevent the development of AKI or minimize its clinical impact. This is important, given that prior studies utilizing automated AKI detection (as opposed to prediction) show limited effectiveness of therapeutic interventions in patients already meeting AKI criteria [26]. We expect that the types of models we develop and validate in the context of this study will have wide-ranging clinical applications.

Our study builds on top of previous studies by integrating the previously identified risk factors for AKI in ICU patients described in the literature including hemodynamic instability, hypoxemia, anemia, inflammation, coagulopathy, liver failure, acidosis, renal/metabolic derangement, and demographics/admission characteristics. In the current study, we investigated the incidence of AKI and the risk factors associated with its development in an ICU population. Our objective was to develop a prediction model capable of discriminating adult patients at high risk of developing new AKI early in their admission to the ICU.

## Methods

### Dataset

This study was a retrospective analysis of critical care patients presenting to the ICU and captured in the Medical Information Mart for Intensive Care III (MIMIC-III). MIMIC-III captures de-identified health information for more than 46,000 patients admitted to the critical care units at Beth Israel Medical Center between 2001 and 2012.

We developed SQL scripts in order to query the MIMIC-III database for all patients who had a creatinine measured at 72 h following ICU admission [27]. We restricted our search to patients 18 years of age or older, and we excluded patients with the pre-existing condition of Chronic Kidney Disease (CKD), who have an estimated GFR (eGFR, MDRD) < 60 mL/min/1.73 m^2^ [28, 29]. Data extracted included patient age, gender, ethnicity, 72-h creatinine, vital signs and lab values during the first day of ICU admission, whether the patient was mechanically ventilated during the first day of ICU admission, and the hourly rate of urine output during the first day of ICU admission.

A total of 23,950 patients met the inclusion criteria for this study. Table 1 presents the predictor variables used in this study, along with their statistical characteristics such as mean and standard deviation. For predictor variables, we excluded those patients who have variables with values that are outside the measurable range (e.g., Glucose value being 999,999). The majority of the patient cohort was male (59.27%), white (72.07%) and the mean age was 60.89 years.

**Table 1:** Univariate Results for Predictors of Interest, *N* = 23,950

Variable	Mean	SD
Gender- N, %		
Female	9755	40.73%
Male	14,195	59.27%
Age (yr)	60.89	16.09
Ethnicity		
African-American	1668	6.96%
White	17,261	72.07%
Hispanic	845	3.53%
Other	4176	17.44%
Creatinine Max during Day 2 and Day 3 (mg/dL)	0.84	0.31
Heart Rate Maximum (bpm)	105.40	19.86
Heart Rate Mean (bpm)	86.97	15.15
Systolic BP Minimum (mmHg)	92.51	16.79
Systolic BP Mean (mmHg)	118.87	15.71
Diastolic BP Minimum (mmHg)	45.31	11.06
Diastolic BP Mean (mmHg)	61.98	10.24
Temperature Maximum (Celsius)	37.60	0.75
SpO2 Minimum (%)	92.23	6.71
SpO2 Mean (%)	97.41	1.85
Glucose Level Maximum (mg/dL)	173.36	74.94
Bicarbonate Level Minimum (mg/dL)	23.87	4.34
Creatinine Level Minimum (mg/dL)	0.74	0.21
Creatinine Level Maximum (mg/dL)	0.80	0.22
Hemoglobin Level Minimum (g/dL)	10.33	2.08
Platelet Count Minimum (K/μL)	210.24	114.82
Potassium Level Maximum (mg/dL)	4.39	0.79
Partial Thromboplastin Time Minimum (s)	32.55	11.72
Partial Thromboplastin Time Maximum (s)	40.22	23.55
International Normalized Ratio Minimum	1.34	0.46
International Normalized Ratio Maximum	1.48	0.70
Prothrombin Time Minimum (s)	14.66	3.61
Prothrombin Time Maximum (s)	15.63	4.95
Blood Urea Nitrogen Level Maximum (mg/dL)	16.87	8.58
White Blood Cell Count Maximum (K/μL)	12.78	9.02
Calcium Level Minimum (mg/dL)	8.08	0.76
Mechanical Ventilation- N, %		
No (0)	11,677	48.76%
Yes (1)	12,273	51.24%
Average Urine Output (mL)	2202.68	1395.37
Estimated Glomerular Filtration Rate (eGFR)	110.20	52.49

*SD* denotes standard deviation

### Statistical analysis

Statistical analysis was performed using R Studio and Python [30, 31]. We ran univariate statistics for patient demographics and the predictors of interest. Univariate linear regression was performed using the maximum creatinine value during Day 2 and Day 3 as the outcome to assess the unadjusted relationship between the predictor of interest and the continuous outcome. Univariate logistic regression was performed using AKI versus no AKI as the outcome. According to KDIGO [10], AKI is defined as either of the following two conditions being met: 1) greater than or equal to 50% increase from the baseline creatinine value to the current creatinine value and 2) greater than or equal to 0.3 mg/dL change in creatinine from the baseline creatinine to the current creatinine value. The pre-ICU baseline creatinine value was defined according to the KDIGO definition by age, race/ethnicity, and gender [10]. We excluded anyone who had AKI on admission (Day 1) because we were interested in predicting new AKI while in the ICU. For AKI status on admission, we compared Day 1 maximum creatinine value (as current creatinine) with pre-ICU baseline. After excluding patients AKI on admission, we then determined the patients’ new AKI status by comparing Day 2 and Day 3 maximum creatinine (as current creatinine) with Day 1 minimum creatinine level.

Our dataset, like most clinical and laboratory datasets obtained in clinical practice, contained missing values, which represent tests not performed in our particular analysis. For example, the variables having a large proportion of missing values include minimum albumin level (74.1%), maximum bilirubin level (67.2%), maximum lactate level (55.8%), maximum c-reactive protein level (99.0%), maximum asparate aminotransferase level (66.8%), maximum pH level (36.6%), and minimum base excess level (64.8%).

Thus, our analysis relies on a two-staged procedure. In the first stage of this procedure, we removed the variables with missing values greater than 20% and we filled in the values for predictors (e.g. labs not performed or recorded) using Multivariate Imputation by Chained Equations (MICE) for those variables with less than 20% missing values. MICE estimates a conditional model for each variable to be imputed, with the other variables as possible predictors [32]. The term chained equation comes from the adoption of a Gibbs sampler, which is an iterative Markov Chain Monte Carlo (MCMC) algorithm for obtaining a sequence of observations that are approximated from a joint probability distribution. As MICE closely track the conditional interdependencies among variables, we expect MICE to produce more accurate imputation. Then, in the second stage, we use the measured and imputed values for these predictors plus age and gender to predict maximum creatinine results during Day 2 and Day 3. In this second stage, we predicted both numerical results for creatinine (linear regression) and whether creatinine increase would be classified as AKI (logistic regression). Although no creatinine results were actually missing from our dataset per the inclusion criteria, we assessed model performance and creatinine predictability by masking creatinine results from a test fold during five-fold cross validation and then compared predicted creatinine results to the masked (measured) values. The masked-measured values were treated as the “ground truth” in assessing model performance. The imputation stage was required because the prediction algorithms used in the second stage of our procedure could not directly accommodate missing data in predictors.

Many lab tests have results that closely follow a log-normal distribution. In linear regression analysis, minimizing the root-mean-square error is equivalent to the maximum likelihood estimation only under the assumption that the target variable adopts a normal distribution. Thus, we transformed creatinine values using a natural log transformation *y* =  *ln* (*x*), where *y* is the transformed creatinine value and *x* is the original creatinine value. The reportable range of creatinine is greater than or equal to 0.1, which guarantees that the log transformation will not produce minus infinity. We inverted this transformation on predicted values of log creatinine (predictions as described below) to calculate predicted values of creatinine in untransformed units.

When performing multivariable linear and logistic regression, all variables with unadjusted relationships with a *p*-value of greater than or equal to 0.05 were excluded from the initial model. Backward selection was used to develop models with all predictors significantly associated with the outcome at a p-value less than or equal to 0.05. One variable, with the highest p-value, was removed from the multivariable model at a time until all remaining variables were significantly associated with the outcome. For linear regression, regression coefficients, 95% confidence intervals, and *p*-values were calculated. For logistic regression, adjusted odds ratios, 95% confidence intervals, and p-values were calculated. A five-fold cross-validation was used to assess the performance of using the selected set or the entire set of variables to predict AKI related outcomes. For linear regression, the root mean square error values were calculated for all 10 runs of the cross-validation and averaged for both the final model developed with backward selection and the model with all predictors included. For logistic regression, the receiver operating characteristic (ROC) curves were developed for the final backward selection model and the model with all predictors of interest. The area under the curve (AUC) values were calculated for both models for all 10 runs and averaged for each model type. Performance metrics (accuracy, sensitivity, specificity, positive predictive value, and negative predictive value) were calculated for the backward selection model and model with all predictors of interest following cross-validation. In addition to logistic regression, we also used other machine learning models including random forests and multilayer perceptron (a type of artificial neural networks) to perform classification. We used the scikit-learn implementations of these machine learning models. For random forest classifier, we set the number of estimators to 100 and max number of tree features to square root of the number of total features. For the multilayer perceptron, we used the Adam solver [33]. As the class ratio is imbalanced, we set the class_weight parameter to “balanced” for logistic regression and random forest classifiers to down weight the more popular class. We also explored the imlearn package for multiple over sampling and under sampling methods including SMOTE [34], ADASYN [35], random under sampling, Tomek Link, and Neighborhood Cleaning Rule. For our task, we found that random under sampling to bring the positive and negative class numbers to comparable level is a simple yet effective method.

## Results

Table 2 presents the bivariate associations between the linear outcome of interest (maximum creatinine during Day 2 and Day 3)/logistic outcome of interest (AKI) and the predictors. Of those included in the study, 3945 (16.5%) total patients had new AKI and 20,005 (83.5%) did not have new AKI according to our definition. In the unadjusted linear regression analyses, minimum diastolic blood pressure, maximum temperature, maximum heart rate, mean heart rate, maximum glucose level, and urine output were not significantly associated with the continuous creatinine outcome and left out of multivariable modeling (*p* > 0.05). In the unadjusted logistic analyses, maximum temperature, minimum international normalized ratio, minimum prothrombin time, maximum heart rate, mean heart rate, maximum glucose level, and urine output were not significantly associated with the dichotomous AKI outcome and left out of multivariable modeling (*p* > 0.05).

**Table 2:** Bivariate associations between linear outcome (maximum creatinine during Day 2 and Day 3) and binary outcome (AKI) and Predictors

Variable	Linear Regression			Logistic Regression		
	Coeff	*P*-Value	95% CI	OR	*P*-Value	95% CI
Gender						
(M: 1,F: 0)	0.2135	< 0.001	[0.206,0.221]	1.23	< 0.001	[1.144,1.318]
Age (yr)	0.0019	< 0.001	[0.002,0.002]	1.02	< 0.001	[1.016,1.020]
Ethnicity						
African-American	ref	ref	ref	ref	ref	ref
Caucasian	−0.0706	< 0.001	[−0.086,-0.055]	1.12	0.106	[0.976,1.294]
Hispanic	−0.1026	< 0.001	[− 0.128,-0.077]	0.73	0.014	[0.566,0.938]
Other	−0.0585	< 0.001	[− 0.076,-0.041]	1.33	< 0.001	[1.136,1.552]
Systolic BP Min (mmHg)	−0.0011	< 0.001	[−0.001,-0.001]	0.98	< 0.001	[0.976,0.980]
Systolic BP Mean (mmHg)	−0.0005	< 0.001	[−0.001,0.000]	0.98	< 0.001	[0.982,0.987]
Diastolic BP Min (mmHg)	−0.0003	0.067	[− 0.001,0.000]	0.98	< 0.001	[0.980,0.986]
Diastolic BP Mean (mmHg)	−0.0009	< 0.001	[−0.001,0.001]	0.98	< 0.001	[0.971,0.978]
Temperature Max (Celsius)	−0.0035	0.190	[−0.009,0.002]	1.04	0.089	[0.994,1.089]
SpO2 Min (%)	−0.0014	< 0.001	[−0.002,-0.001]	0.99	< 0.001	[0.983,0.991]
SpO2 Mean (%)	−0.0028	0.010	[−0.005,-0.001]	1.08	< 0.001	[1.055,1.098]
Bicarbonate Level Min (mg/dL)	−0.0049	< 0.001	[−0.006,-0.004]	0.94	< 0.001	[0.934,0.949]
Creatinine Level Minimum (mg/dL)	0.9479	< 0.001	[0.933,0.963]	–	–	–
Creatinine Level Max (mg/dL)	0.9340	< 0.001	[0.921,0.947]	3.20	< 0.001	[2.743, 3.737]
Hemoglobin Level Min (g/dL)	−0.0048	< 0.001	[− 0.007,-0.003]	0.77	< 0.001	[0.759,0.787]
Platelet Count Min (K/μL)	− 0.0004	< 0.001	[− 0.000,0.000]	1.00	< 0.001	[0.996,0.997]
Potassium Level Max (mg/dL)	0.0837	< 0.001	[0.079,0.089]	1.90	< 0.001	[1.824,1.984]
Partial Thromboplastin Time Min (s)	0.0020	< 0.001	[0.002,0.002]	1.01	< 0.001	[1.010,1.015]
Partial Thromboplastin Time Max (s)	0.0012	< 0.001	[0.001,0.001]	1.01	< 0.001	[1.008,1.010]
International Normalized Ratio Min	0.0213	< 0.001	[0.013,0.030]	1.04	0.266	[0.970,1.118]
International Normalized Ratio Max	0.0274	< 0.001	[0.022,0.033]	1.21	< 0.001	[1.158,1.260]
Prothrombin Time Min (s)	0.0032	< 0.001	[0.002,0.004]	1.01	0.194	[0.997,1.015]
Prothrombin Time Max (s)	0.0042	< 0.001	[0.003,0.005]	1.03	< 0.001	[1.021,1.033]
Blood Urea Nitrogen Level Max (mg/dL)	0.0088	< 0.001	[0.008,0.009]	1.01	< 0.001	[1.009,1.017]
White Blood Cell Count Max (K/μL)	0.0008	< 0.001	[0.000,0.001]	1.01	< 0.001	[1.010,1.018]
Calcium Level Min (mg/dL)	0.0245	< 0.001	[0.019,0.030]	0.72	< 0.001	[0.685,0.749]
Mechanical Ventilation (Yes 1/No 0) - N, %	0.0618	< 0.001	[0.054,0.070]	3.04	< 0.001	[2.816,3.275]
Estimated Glomerular Filtration Rate (eGFR)	−0.0025	< 0.001	[−0.003,-0.002]	1.00	< 0.001	[1.004,1.005]

*CI* denotes confidence interval, *OR* denotes odds ratio, Coeff denotes linear regression coefficient. The regression coefficient and odds ratio are unadjusted regression coefficient and odds ratio respectively

The multivariable linear regression model results are presented in Table 3. All predictors were significantly associated with maximum creatinine during Day 2 and Day 3. The multivariable logistic regression model results are presented in Table 4. All predictors were significantly associated with AKI. Comparing the two tables, most of the significant AKI predictors are similar to those found in the linear regression. These predictors are consistent with the known pathophysiology of AKI. Males, older patients, and African-Americans have higher incidence of AKI. Hypoxemia (low SpO2), mechanical ventilation, and coagulopathy (prolonged partial thromboplastin and prothrombin times) are also known risk factors of AKI and also might represent patients with higher severity of illness. Patients with less anemia (high hemoglobin) are probably less likely to have AKI due to the increased oxygen carrying capacity and the lower likelihood of requiring a blood transfusion, which is a known risk factor of AKI. The “protective” effect of high blood urea nitrogen level (and possibly high hemoglobin) probably represents patients with intravascular hypovolemia who are less likely to be diagnosed with AKI after the dilutional effect of fluid resuscitation. The elevated potassium and calcium levels likely represent early electrolyte disturbances in the setting of injured kidneys likely to meet AKI definition in the subsequent days. Finally, the elevated creatinine level, while not meeting AKI criteria in these patients given the exclusion criteria of the study, likely represents an early elevation indicative of injured kidneys in which the creatinine level hasn’t peaked yet. Note that the high bicarbonate level, which was not selected in the linear regression, but achieved a small significance level in the logistic regression, likely represents a surrogate of less acidosis, which is associated with higher severity of illness and is a risk factor of AKI.

**Table 3:** Multivariable linear regression results following backward selection

Variable	Adjusted Regression Coefficient	*P*-Value	95% Confidence Interval
Gender			
(M: 1,F: 0)	0.026	< 0.001	[0.019,0.034]
Age	0.020	< 0.001	[0.016,0.023]
SpO2 Mean	−0.013	< 0.001	[− 0.016,-0.009]
Bicarbonate Level Minimum	−0.005	0.005	[−0.008,-0.001]
Creatinine Level Minimum	0.364	< 0.001	[0.326,0.403]
Creatinine Level Maximum	0.137	< 0.001	[0.130,0.144]
Hemoglobin Level Minimum	−0.024	< 0.001	[− 0.027,-0.021]
Platelet Count Minimum	−0.018	< 0.001	[− 0.021,-0.015]
Potassium Level Maximum	0.021	< 0.001	[0.018,0.025]
Partial Thromboplastin Time Minimum	0.016	< 0.001	[0.013,0.019]
International Normalized Ratio Minimum	−0.025	< 0.001	[− 0.035,-0.015]
International Normalized Ratio Maximum	0.016	< 0.001	[0.011,0.020]
Blood Urea Nitrogen Level Maximum	−0.014	< 0.001	[−0.017,-0.011]
Calcium Level Minimum	0.014	< 0.001	[0.011,0.017]
Mechanical Ventilation	0.018	< 0.001	[0.015,0.021]
Estimated Glomerular Filtration Rate	0.009	< 0.001	[0.004,0.013]

**Table 4:** Multivariable logistic regression results following backward selection

Variable	Adjusted Odds Ratio	*P*-Value	95% Confidence Interval
Gender			
(M: 1,F: 0)	0.59	< 0.001	[0.553,0.647]
Age	1.56	< 0.001	[1.494,1.635]
Ethnicity			
African-American	ref	ref	ref
Caucasian	1.47	< 0.001	[1.248,1.721]
Hispanic	1.12	0.414	[0.850,1.484]
Other	1.65	< 0.001	[1.382,1.970]
Systolic BP Minimum	0.94	0.003	[0.897,0.977]
SpO2 Mean	0.89	< 0.001	[0.852,0.925]
Bicarbonate Level Minimum	0.90	< 0.001	[0.858,0.938]
Creatinine Level Maximum	2.68	< 0.001	[2.501,2.862]
Hemoglobin Level Minimum	0.74	< 0.001	[0.711, 0.776]
Platelet Count Minimum	0.80	< 0.001	[0.767,0.839]
Potassium Level Maximum	1.28	< 0.001	[1.236,1.334]
Partial Thromboplastin Time Minimum	1.12	< 0.001	[1.078,1.158]
International Normalized Ratio Maximum	1.06	< 0.001	[1.025,1.099]
Blood Urea Nitrogen Level Maximum	0.89	< 0.001	[0.857,0.933]
Calcium Level Minimum	1.08	< 0.001	[1.034,1.130]
Mechanical Ventilation	1.45	< 0.001	[1.386,1.519]
Estimated Glomerular Filtration Rate	2.93	< 0.001	[2.734,3.140]

Due to the nature of random fold split in cross-validation, we ran both cross-validated linear regression and logistic regression 10 times with different random seeds to account for performance variability. Table 5 presents the regression results from the cross-validation for linear regression. Table 6 presents the classification results from the cross-validation using logistic regression, random forests, and multilayer perceptron. For linear regression, the backward selection model had the same root mean square error value than the model with all variables (0.224 vs. 0.224 mg/dL, respectively). In MIMIC-III dataset, the serum creatinine level is reported to 0.1 mg/dL, with a range 10 mg/dL. Thus, the root mean square error should be considered low both in absolute value and in percentage over range (2.2%). Although the RMSE is close to the 0.3 mg/dL change in the KDIGO AKI criteria, our logistic regression model designed to predict AKI categorical status achieved competitive performance. For logistic regression, the backward selection model had a lower area under the curve than the model with all variables (0.780 vs. 0.783, respectively). For random forests, the backward selection model had a lower area under the curve than the model with all variables (0.772 vs. 0.779, respectively), both lower than logistic regression. Multilayer perceptron gives the best performance with the backward selection model all variables models having AUCs of 0.792 and 0.796 respectively. Given that most previous models had modest AUCs around 0.75 (see Related Work section), our model gives a better performance than previous models when using all-variable models. In addition, our results generalize to much larger general adult critical care patient cohort. Table 7 presents the performance metrics for the logistic regression models. Across all machine learning algorithms and both backward selected and all-variable models, recall (sensitivity: 0.660–0.698) is favored over precision (positive predictive value: 0.337–0.357). This conforms to the clinical need in that given the relative low prevalence and high stake of AKI, one wants to catch as many AKI cases as possible and is willing to bear with false alarms.

**Table 5:** Linear regression results from the cross-validation

	Root Mean Square Error Values										
	Mean	Run 1	Run 2	Run 3	Run 4	Run 5	Run 6	Run 7	Run 8	Run 9	Run 10
Linear Backward Selection Model	0.224	0.224	0.224	0.224	0.224	0.224	0.224	0.224	0.224	0.224	0.224
Linear All Variables Model	0.224	0.224	0.224	0.223	0.224	0.224	0.224	0.223	0.224	0.224	0.224

**Table 6:** Machine learning results from the cross-validation

	Area Under the Curve Values										
	Mean	Run 1	Run 2	Run 3	Run 4	Run 5	Run 6	Run 7	Run 8	Run 9	Run 10
LR Backward Selection Model	0.780	0.780	0.780	0.779	0.780	0.780	0.780	0.780	0.780	0.780	0.780
LR All Variables Model	0.783	0.782	0.782	0.782	0.782	0.782	0.782	0.782	0.782	0.782	0.783
RF Backward Selection Model	0.772	0.772	0.774	0.775	0.772	0.773	0.773	0.773	0.775	0.773	0.772
RF All Variables Model	0.779	0.779	0.779	0.780	0.779	0.780	0.778	0.777	0.780	0.779	0.779
MLP Backward Selection Model	0.792	0.794	0.794	0.791	0.792	0.793	0.794	0.792	0.793	0.794	0.792
MLP All Variables Model	0.796	0.796	0.798	0.796	0.795	0.796	0.796	0.794	0.795	0.796	0.796

**Table 7:** Other average performance metrics from the cross-validation

	Accuracy	Sensitivity	Specificity	Positive Predictive Value	Negative Predictive Value
LR Backward Selection Model	0.724	0.697	0.730	0.337	0.924
LR All Variables Model	0.729	0.698	0.736	0.342	0.925
RF Backward Selection Model	0.739	0.660	0.754	0.346	0.918
RF All Variables Model	0.742	0.673	0.756	0.352	0.921
MLP Backward Selection Model	0.744	0.684	0.756	0.356	0.924
MLP All Variables Model	0.743	0.694	0.753	0.357	0.926

**Fig. 1:**
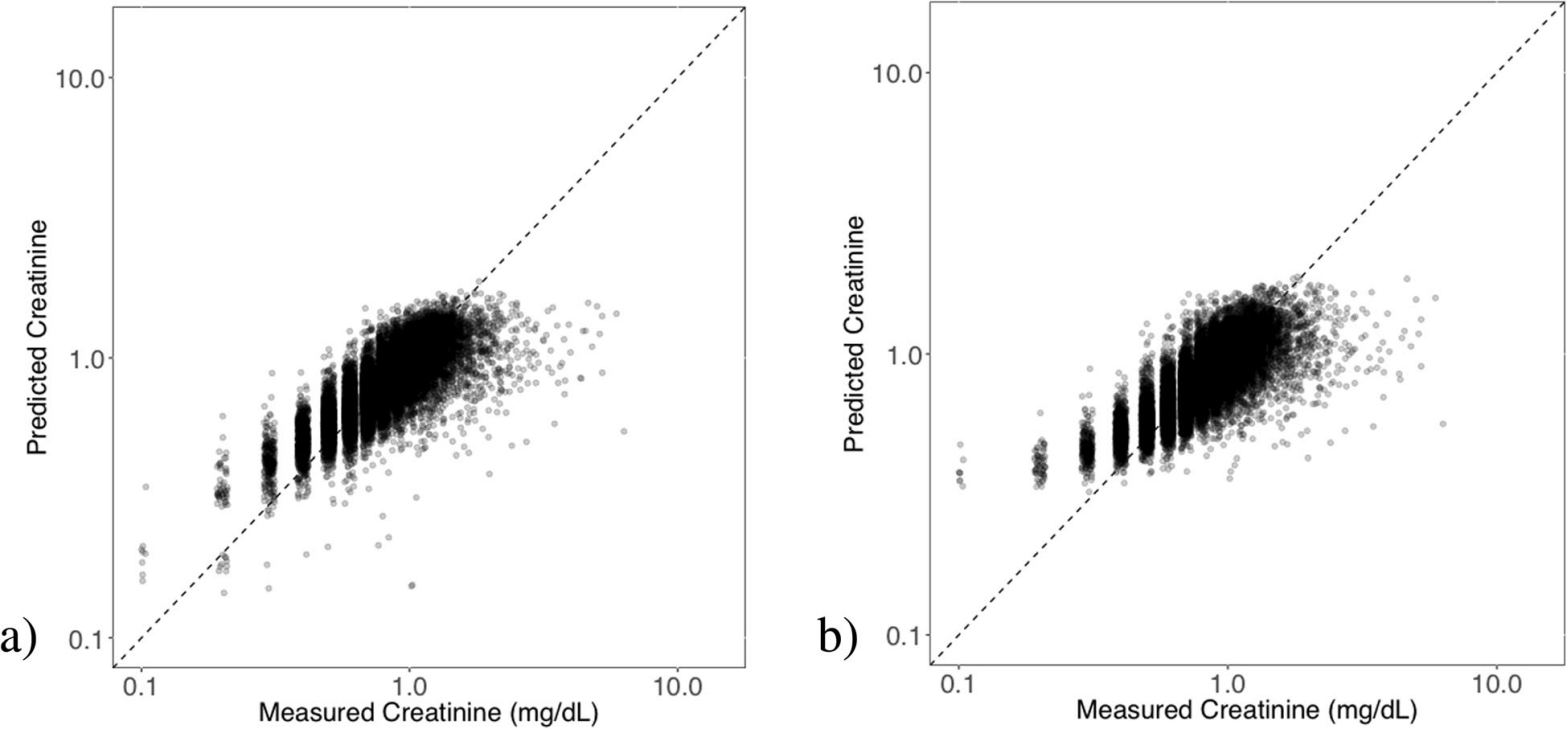
Scatter plots comparing the measured and predicted creatinine values using linear regression models with **a**) backward selection variable model and **b**) all variables model after cross-validation. Note that the axes are on a logarithmic scale or plot log transformed data

**Fig. 2:**
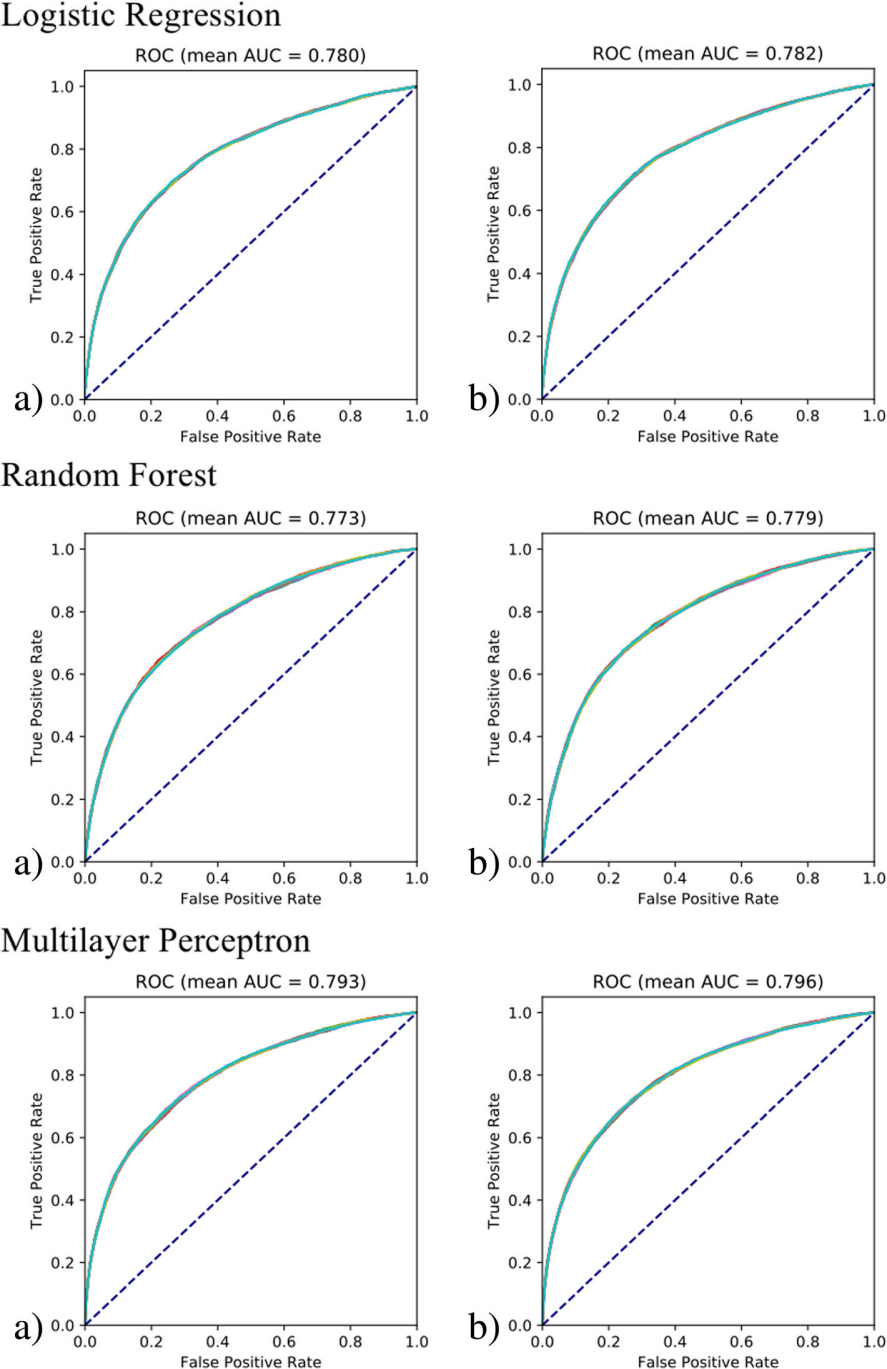
ROC curves for logistic regression, random forest, and multilayer perceptron models using **a**) backward selection model and **b**) all-feature model using cross-validation. We repeat the 5-fold cross validation 10 times, each time using stratified 5-fold split with different random initializations. We use different colors for ROC curves from different cross validations. Note that for both for both the all-variable and backward selection models, the model performance is insensitive to stratified 5-fold splits with different random initializations. Thus, the ROC curves are almost identical to each other

Next, we more closely investigated the extent to which serum creatinine can be predicted. We examined scatter plots denoting the relationship between measured and predicted creatinine values, as shown in Fig. 1. Both models generated similar prediction plots. Note that predictions were most accurate toward the middle of the dynamic range of measured creatinine results, presumably due to “regression toward the mean.” Some of the numerical prediction error may thus be attributed to measured creatinine values toward the high end of the range with predicted values of creatinine that are considerably lower, but that are still within the reference range. Furthermore, the regression algorithms in general train to minimize error throughout the dynamic range, thus explaining the behavior in the plots. Figure 2 further plots the ROC curves (overlay of all 10 runs) for classifying AKI binary outcome with the logistic regression, random forest, and multilayer perceptron models using variables from backward selection and all variables. For logistic regression, the two models exhibited different classification performance, which is evident from similar AUCs and ROC curves. The backward selection model gives an averaged AUC of 0.780 comparable to previous systems and is insensitive to different cross validation random splits. The all-feature model gives a higher averaged AUC of 0.783. The fact that both the linear regression and the logistic regression backward selection models are not sensitive to cross validation splits likely suggests the robustness of the model and the predictability of both the numeric serum creatinine level and the binary AKI classification. Thus we chose the backward selection model when evaluating the individual factor association with the serum creatinine value and new AKI status as a conservative, but reliable way to derive clinical insights. On the other hand, the higher AUCs from all-feature model suggest the effectiveness and potential of an entirely data-driven approach over a more parsimonious model. The fact that all-feature model subjects to more randomness in the data likely calls for a more effective feature selection method that takes statistical stability into consideration.

## Discussion

In our two-stage analysis, we have used MICE imputation to fill in missing values for each variable. This approach imputes missing values by the MICE algorithm [32, 36]. Theoretically, this method is only unbiased under the assumption that the data is missing-at-random. Real clinical practice surely violates this assumption since clinicians usually order tests given some expectations about the likely results. Nonetheless, our previous study shows that imputation bias due to not missing-at-random may have only minimal impact on patient outcome prediction [37]. In practice, it may be used as an effective way and baseline for comparing other multiple imputation methods due to its simple implementation [38]. We are also aware of the limitations of imputation for clinical data, as missingness may represent no indication for having the test performed. To this end, we will also investigate missingness patterns as predictors in future studies.

We recognize a number of additional limitations of our initial study. Our models were primarily based on demographic, vital signs, and laboratory data but did not factor in comorbid diagnoses. This is similar to other predictive scores of severely ill patients such as the MELD [39] and APACHE II [40] scoring systems, which are also based on well-defined structured laboratory or vital signs data. In the ICU setting, patients often suffer from multiple acute upon chronic conditions where the acute immediate condition may play a more important role than prior chronic conditions, hence the relatively larger impact of current laboratory tests or vital signs. However, future work will include comorbid diagnoses as factors in multivariate regression analyses. Conditions which affect the kidneys, such as diabetes, may be disproportionate in their effect, and will need to be accounted for in future work.

In this study, we also focused on predicting AKI using first-day measurements of a multivariate panel of physiologic variables but not medications and procedures, in order to elucidate early, subclinical deterioration of patients’ physiologic baselines that are predictive of AKI. We also focused on establishing the baseline of using physiologic variables to predict AKI with large cohort size but did not consider dynamic prediction window. The Kidney Disease Improving Global Outcomes (KDIGO) clinical practice guidelines examines serum creatinine change within 48 h [13]. We will examine in future studies the AKI prediction with physiologic and medication data and with dynamic prediction windows (e.g., using patient data up to Day n to predict AKI in Day n + 1 and Day n + 2). We also plan to explore the temporal changes of the model predictors to improve the prediction model performance.

In the backward linear and logistic regression models, predictors included did vary. Included in linear regression and not in logistic regression were the following variables: creatinine level minimum and international normalized ratio minimum. Ethnicity and systolic blood pressure minimum were the only variables included in logistic regression that was not included in linear regression. As it is uncommon for the linear regression model to use more predictors than the logistic regression model due to more detailed outcome prediction (numeric vs. binary label) by the former, we will use clinical content experts to suggest a pared down list of predictors or natural clinically relevant cutoff scores for variables, to see if these will improve model performance and relevance. When selecting the predictors, in addition to using the *p*-values as the sole criteria, we plan to integrate other factors such as effect size as additional criteria during predictor selection in the future.

## Conclusions

We demonstrated the feasibility of developing a model for early prediction of AKI in the first 72 h following ICU admission in an adult patient population in critical care setting. Through cross-validation on linear regression and multiple machine learning models, we showed that comprehensive demographics and physiologic features can accurately predict max serum creatinine level during Day 2 and Day 3 with a root mean square error of 0.224 mg/dL. The same demographics and physiologic features can also predict new AKI onset as defined by the current clinical guideline with a competitive AUC (mean AUC 0.783 by our all-feature model), while previous models aimed at more specific patient cohorts. Our work suggests that prospective trials with independent model training and validation cohorts are needed to further evaluate the clinical utility of this approach for identifying at risk patients early in their hospital course and potentially instituting interventions to decrease the likelihood of developing AKI.

## Abbreviations

AKI: Acute kidney injury
AUC: Area under the receiver operating curves
CKD: Chronic Kidney Disease
eGFR: estimated glomerular filtration rate
EHRs: Electronic health records
ICU: Intensive care unit
KDIGO: Kidney Disease Improving Global Outcomes
MCMC: Markov Chain Monte Carlo
MICE: Multivariate Imputation by Chained Equations
MIMIC-III: Medical Information Mart for Intensive Care III
NGAL: Neutrophil gelatinase-associated lipocalin
RMSE: Root-mean-square error
ROC: Receiver operating characteristic
SCr: Serum creatinine
